# Trajectories of Social Anxiety during Adolescence and Relations with Cognition, Social Competence, and Temperament

**DOI:** 10.1007/s10802-012-9651-6

**Published:** 2012-06-22

**Authors:** A. C. Miers, A. W. Blöte, M. de Rooij, C. L. Bokhorst, P. M. Westenberg

**Affiliations:** 1 Faculty of Social and Behavioral Sciences, Institute of Psychology, Unit Developmental and Educational Psychology, Leiden University, Pieter de la Court building, P.O. Box 9555, 2300 RB Leiden, the Netherlands; 2 Faculty of Social and Behavioral Sciences, Institute of Psychology, Unit Methodology and Statistics, Leiden University, Pieter de la Court building, P.O. Box 9555, 2300 RB Leiden, the Netherlands

**Keywords:** Trajectories of social anxiety, Social skills, Negative cognitions, Neuroticism

## Abstract

This cohort-sequential study examined developmental trajectories of social anxiety in a nonclinical sample (*N* = 331, 161 girls) aged 9 to 17 years at initial and 12 to 21 years at final assessment. We tested whether variables assessing cognition, social competence, and temperament discriminated between the trajectories. Variables were collected from different sources: participants, independent observers, parents, and teachers. Using Latent Class Growth Modeling (LCGM) we identified three distinct social anxiety trajectory groups: i) high and changing; ii) moderate and decreasing; and iii) low and decreasing. Multinomial regression analyses showed that the cognition variables, negative interpretations of ambiguous social situations and self-focused attention, differentiated between all three trajectories. A lack of social skills and having social problems at school were specifically related to the chance of following the high trajectory versus the moderate trajectory. Neuroticism differentiated between the low and moderate trajectories. Findings indicate that adolescents at risk of belonging to a high social anxiety trajectory can be discriminated from peers belonging to a less anxious trajectory using both cognition and social competence variables.

In their frequently cited review of the literature on social phobia (or Social Anxiety Disorder, SAD), Rapee and Spence (2004) opened with the message that whilst the field has moved forward in understanding factors that may maintain social phobia, it is still relatively unclear which variables bring about individual differences in social anxiety levels in the population. A first step that needs to be taken before these causal factors are addressed is to capture individual differences in the development of social anxiety over time with longitudinal studies. The longitudinal study described in this article explored different developmental trajectories of social anxiety in a nonclinical sample, spanning adolescence through emerging adulthood, using the growth mixture modeling technique. Second, we examined whether conceptually relevant cognition, social competence, and temperament variables could discriminate between trajectories of social anxiety. In the present study we use the term ‘social anxiety’ to refer to a continuum of social anxiety ranging from low to high and including its clinical form, social phobia (Rapee and Spence 2004; Rapee and Heimberg 1997). In discussing previous literature we specify whether studies focused on nonclinical samples with high levels of social anxiety symptoms or clinical samples with the diagnosis of social phobia.

Adolescence seems to be a key period in the developmental course of social anxiety. Social phobia generally has its onset in the early to mid teens, between 10 and 13 years (Rapee and Spence 2004). Although high levels of social anxiety can occur in childhood too, a number of changes that take place in early to mid adolescence may contribute to the emergence of social phobia specifically in the adolescent years; for example, physical changes that accompany puberty, socio-cognitive maturation, changes in the school environment, and the increasing importance of social interactions with same age peers (Inderbitzen-Nolan and Walters 2000; Rapee and Spence 2004; Westenberg et al. 2004). High levels of social anxiety in childhood may thus become more problematic in early adolescence. Research with nonclinical samples reveals a normative increase in the sensitivity toward negative social evaluation in mid to late adolescence or 15 to 18 years (Sumter et al. 2010; Westenberg et al. 2004). In addition, the continuity of individual differences in social evaluation fears increases from pre to late adolescence (Westenberg et al. 2007). In an adult community sample, substantial continuity in high levels of social anxiety symptoms was found in individuals aged between 20 and 35 years (Merikangas et al. 2002). Hence, the adolescent period is a crucial phase for tracking social anxiety differences among individuals over time and investigating whether individuals can be grouped according to their particular developmental trajectory.

## Trajectories of Social Anxiety in Children and Adolescents and Related Variables

The practice of identifying subgroups of people who follow distinct developmental trajectories of internalizing problems using the growth mixture modeling technique is growing steadily and there is evidence for heterogeneous groups during childhood and adolescence. To date trajectories of anxiety have mostly been described in child and preadolescent populations and based on parent or teacher report of anxiety symptoms (Broeren et al. 2011; Duchesne et al. 2010; Feng et al. 2008; Marmorstein et al. 2010). Three of these studies (Broeren et al. 2011; Duchesne et al. 2010; Feng et al. 2008) found four trajectories of anxiety or social anxiety in a total age range of 2 to 12 years. The trajectory patterns of anxiety were fairly similar with low, low increasing, high declining, and high stable or high increasing groups (Duchesne et al. 2010; Feng et al. 2008) whereas the groups for social anxiety were stable low, medium, high, and very high (Broeren et al. 2011). Marmorstein et al. (2010) identified five trajectories of parent reported social anxiety over the age range 5 to 13 years: low increasing, stable moderate, moderate decreasing, high decreasing, and stable high. Both studies on social anxiety thus found two stable trajectories, moderate or medium and high. The three trajectories showing change over childhood in the Marmorstein et al. (2010) study as compared to stable patterns reported by Broeren et al. (2011) could possibly be explained by method and sample differences, such as sample size, gender composition, and age range. For example, Marmorstein et al.’s (2010) sample of over 2,000 participants was ten times as large as Broeren et al.’s (2011) sample, only consisted of girls, and was older in age.

Only one study has investigated anxiety trajectories in an adolescent to emerging adulthood sample. Crocetti et al. (2009) found two trajectories of self-reported anxiety symptoms in adolescence, a low and decreasing and a high and increasing one. These two trajectories were found for an early adolescent cohort, aged between 10 and 15 years at the first assessment and a middle adolescent cohort, aged between 16 and 20 years at first assessment. Both cohorts were measured annually for 5 years.

In sum, trajectory studies point to four or five heterogeneous groups for either generalized or social anxiety symptoms when studied in childhood to preadolescence. One study shows two groups for generalized anxiety in the adolescent to emerging adulthood period. However, none of these studies examined potential subgroups of social anxiety over the whole adolescent period despite the relevance of this developmental phase to the increase of social anxiety in nonclinical populations (Westenberg et al. 2004). It is important to identify potential subgroups of adolescents with differing levels of social anxiety over time because with this information a richer analysis of potential variables that are associated with continuity or emergence of social anxiety during adolescence is possible. In this study we examined three individual level variables, namely cognition, social competence, and temperament, which are theoretically and empirically related to social anxiety and may discriminate between social anxiety trajectories. Our choice of variables was partly motivated by the seminal review of the etiology of social phobia by Rapee and Spence (2004).

### Cognition and Social Anxiety

The current study includes three cognition variables selected from cognitive models of social phobia (Clark and Wells 1995; Rapee and Heimberg 1997), namely negative interpretations, self-focused attention, and self-evaluation of performance.

Negative interpretations refers to the tendency to negatively interpret ambiguous cues in social situations and is termed an interpretation bias (Clark and Wells 1995; Miers et al. 2008). Several studies show that adults and adolescents with social phobia or high levels of social anxiety are more likely to interpret ambiguous social cues in a negative manner than nonanxious persons (see Miers et al. 2011b for a review). Moreover, the interpretation bias appears to be particular to social anxiety rather than a general feature of emotional disorders such as depression (Miers et al. 2011b). Vassilopoulos et al. (2009) also showed that by training high socially anxious preadolescents to make more benign interpretations of ambiguous social cues, social anxiety symptoms decrease. Hence, in line with the cognitive models it is well established that high social anxiety and social phobia are associated with negative interpretations of ambiguous social cues.

Self-focused attention is the allocation of one’s attentional resources to internal aspects such as arousal, behavior, thoughts, and emotions (Bögels and Mansell 2004). The Clark and Wells (1995) model states that when a social phobic person enters a feared social situation their attentional focus shifts to the self. This self-focus is said to interfere in the processing of external social cues and attending to the situation at hand. There is abundant evidence in the adult literature to show that socially anxious persons or patients with social phobia report more self-focused attention than nonanxious persons during social situations (e.g., Bögels and Mansell 2004) and a growing number of studies confirm this cognitive process in nonclinical youth samples (e.g., Hodson et al. 2008). Some, but not all, studies in which attentional focus is experimentally manipulated also provide evidence for the link between self-focused attention and elevated social anxiety or social phobia (see Schultz and Heimberg 2008 for a review).

The third cognition variable, self-evaluation of social performance, is grounded in socially anxious persons’ beliefs, such as that other people are inherently critical and holding excessively high standards for social performance (Clark and Wells 1995; Rapee and Heimberg 1997). As a result of these beliefs socially anxious persons negatively evaluate their own performance and behavior during social situations. There is ample evidence, from clinical and nonclinical samples, that after engaging in a social situation or task (e.g., conversation or speech) socially anxious adults (e.g., Norton and Hope 2001) and youth (e.g., Inderbitzen-Nolan et al. 2007; Miers et al. 2009) have more negative self-evaluations than their nonanxious counterparts.

In sum, the available evidence suggests that negative interpretations and self-evaluation of social performance have strong associations with social anxiety in cross-sectional studies, with weaker evidence for the link between self-focused attention and social anxiety in youth. It is still unclear to what extent these cognition variables may longitudinally differentiate between trajectories of social anxiety during adolescence (Muris 2010).

### Social Competence and Social Anxiety

Accumulating evidence from clinical and nonclinical samples shows that socially anxious youth are more rejected and less liked by, and receive more negative treatment from peers than their nonanxious counterparts (e.g., Blöte et al. 2007; Spence et al. 1999; Verduin and Kendall 2008). One reason for receiving negative treatment is that socially anxious youth are less socially competent than their nonanxious peers. As defined by Spence et al. (1999) poor social competence may be evident in immediate, first time judgments of a person’s social performance and in long-term outcomes of social interactions. The current study included nervousness and social skills as social performance variables, and social problems at school as an indicator of social competence in long-term interactions.

Laboratory studies measuring social performance during different types of social tasks include both the actual skills (e.g., eye contact, posture, etc) employed during a social situation and overt nervousness (i.e., visible signs of anxiety like blushing, stuttering, and general nervous appearance; Miers et al. 2009). Quite a number of these studies show that in clinical and nonclinical samples socially anxious adults and youth receive poorer evaluations of their social skills and appear more nervous than their nonanxious counterparts, as rated by independent observers (e.g., Inderbitzen-Nolan et al. 2007; Norton and Hope 2001). However, some studies do not find evidence for social skills deficits, as rated by adult observers, in socially anxious youth (e.g., Cartwright-Hatton et al. 2005).

Inadequate social competence has also been linked to (social) anxiety in studies that assess behavior in the context of, or with reference to, the natural environment, for example at school or interactions with friends. Spence et al. (1999) compared a group of social phobic children with a control group (age range 7–14 years) on a measure of social competence with peers, as rated by a parent, and during natural observation of their interactions with peers at school. According to both parents and observers children in the social phobia group were less socially competent with their peers and elicited fewer positive responses from peers than children in the control group. Schneider (2009) examined social behavior in the context of close friendships and showed that a referred, but not clinical, sample of socially withdrawn, anxious children aged 10 to 12 years were unassertive, uncommunicative and displayed little positive affect.

The literature to date has shown the importance of poor social competence in relation to social anxiety in terms of both first time judgments and as an outcome of long-term interactions. In this study we will examine the longitudinal contribution of each type of social competence, that is, nervousness and social skills during a social task and social problems at school, to different patterns of social anxiety during adolescence and emerging adulthood.

### Temperament and Social Anxiety

The third category of variables that may be related to trajectories of social anxiety is temperament. Based on the literature we selected four variables, neuroticism, extraversion, behavioral inhibition, and social withdrawal.

The Big Five personality theory is a widely accepted model (McCrae and John 1992) that captures individual differences in five traits: agreeableness, neuroticism, extraversion, conscientiousness, and openness (Watson et al. 1994). Of these five, two have been linked to (social) anxiety, namely neuroticism and extraversion, both of which have been referred to as dimensions of temperament (Watson et al. 2005). Neuroticism is characterized by a trait sensitivity to negative stimuli and a tendency to experience unpleasant emotions; extraversion is the tendency to experience positive emotions and be sociable (Watson et al. 1994). It is widely accepted that neuroticism is positively related to anxiety in general and it has also been specifically linked to social phobia in adults (Bienvenu and Stein 2003) and youth (e.g., Beidel et al. 1999). Social anxiety is associated with low extraversion in clinical (Beidel et al. 1999; Bienvenu and Stein 2003) and nonclinical samples (Watson et al. 2005).

Behavioral inhibition and its conceptually related construct, social withdrawal, are also commonly associated with social anxiety. Behavioral inhibition is defined as “the disposition to be wary and fearful when encountering novel (that is, unfamiliar) situations” (Burgess et al. 2001; p.3) whereas social withdrawal refers to solitary behavior not only in unfamiliar but also *familiar* situations, for example with one’s peers (Burgess et al. 2001). Longitudinal studies investigating behavioral inhibition in young children indicate that it is a risk factor for the development of anxiety and depressive disorders (see Degnan et al. 2010 for a review). Some studies suggest that behavioral inhibition is a risk factor for social phobia specifically (e.g., Chronis-Tuscano et al. 2009), although not all children identified as inhibited go on to develop social phobia. Similarly, social withdrawal in childhood is a risk factor for developing high levels of anxiety (e.g., Ladd 2006).

In sum, there is considerable empirical evidence for a relationship between social anxiety and these four aspects of temperament, but they have not been examined together in relation to different trajectories of social anxiety before.

## Present Study

The present cohort-sequential longitudinal study addressed two research questions: (1) is it possible to identify distinct longitudinal trajectories of social anxiety across the adolescent and emerging adulthood period, 9 to 21 years? And (2) which of the cognition, social competence, and temperament variables discriminate between the social anxiety trajectories? We used data from the Social Anxiety and Normal Development (SAND; Westenberg et al. 2009) study to investigate these research questions. The SAND study includes four assessment waves with participants aged between 9 and 17 years at Wave 1 and between 12 and 21 years at Wave 4. The Latent Class Growth Modeling (LCGM; Nagin 2005) technique, which has previously been used with this type of longitudinal design (Marmorstein et al. 2010), was employed to identify trajectories of social anxiety.

Gender differences are often found on self-report measures of social anxiety symptoms (Rapee and Spence 2004) and for at least some of the other variables in the present study, for example negative interpretations (Miers et al. 2008) and social skills (Miers et al. 2009). Our sample size was not large enough to test whether boys and girls possibly follow different social anxiety trajectories. However, we tested for differences in the number of boys and girls in the social anxiety trajectory groups, checked for gender differences on the cognition, social competence and temperament variables and controlled for gender in the analyses differentiating social anxiety trajectory groups on the basis of these variables.

In relation to the first research question, because the available studies to date have mainly dealt with a different type of anxiety and/or investigated a different age group (Broeren et al. 2011; Duchesne et al. 2010; Feng et al. 2008; Marmorstein et al. 2010) it was difficult to formulate a hypothesis regarding the number of trajectories of social anxiety. Nevertheless, as an indication we drew from the Crocetti et al. (2009) study because it has a comparable age range and describes trajectories of overall self-reported anxiety, including social anxiety symptoms. Based on this study we expected to find at least two different trajectories of social anxiety, with a high increasing group and one or more groups with lower social anxiety. As regards the second research question, we expected that all three categories of variables, cognition, social competence, and temperament, would differentiate a high trajectory of social anxiety from one or more trajectories of lower social anxiety. We expected negative interpretations, self-focused attention, nervousness, social problems, neuroticism, behavioral inhibition, and social withdrawal to be positively associated with a high social anxiety trajectory whereas self-evaluation of performance, social skills, and extraversion would be negatively associated.

## Method

### Participants

Participants were drawn from the SAND longitudinal study (Westenberg et al. 2009) that included children and adolescents recruited from one secondary school and two primary schools in an urban area of the Netherlands. At the first wave (W1) the total sample comprised 331 participants (170 boys and 161 girls) and had a mean age of 13.34 years (*SD* = 2.25). The mean social anxiety sum score in the total sample was 40.67 (*SD* = 12.75) at W1, which is comparable with normative levels reported in previous studies (Inderbitzen-Nolan and Walters 2000; La Greca and Lopez 1998) and less than the mean in a clinical sample of adolescents aged 12 to 17 years (La Greca 1999). Eighty-two (81.6 %) percent of participants lived with biological parents, 5.7 % with biological mother only and 5.1 % with biological mother and stepfather. Ninety-two (91.5 %) percent of participants were born in the Netherlands and 49.0 % of biological mothers had completed tertiary education.

The retention rate from wave to wave ranged between 83 % and 95 %. The number of participants per wave was 331, 298, 248, and 236, respectively. At W4 there were 121 boys and 115 girls with a mean age of 17.48 years (*SD* = 2.72). Informed parental consent and assent from participants themselves was obtained in writing at each study wave. The SAND study was approved by the University’s Medical Ethical Committee.

We examined missing data according to the number of missing responses per wave on our primary variable, social anxiety. The number of participants with 1, 2, 3 or 4, valid responses was, respectively, 30, 54, 18 and 229. Two regression analyses were conducted, a logistic regression with a valid response at W4 versus no response at W4 as a dichotomous outcome variable, and a multiple regression with total number of missing responses across all waves as a continuous outcome variable. These analyses showed that missing data were independent of W1 age and social anxiety, as well as gender and all other variables used in this study (*χ* ^2^(13) = 15.89, *ns*; *F*(13) = 1.08, *ns*). Because the study design is cohort-sequential, there was a different number of participants with a valid response on the social anxiety measure in each age cohort from 9 to 21 years (9 years, *n* = 26; 10 years, *n* = 67, 11 years *n* = 101, 12 years *n* = 107, 13 years *n* = 131, 14 years *n* = 134, 15 years *n* = 152, 16 years *n* = 124, 17 years *n* = 110, 18 years *n* = 65, 19 years *n* = 39, 20 years *n* = 33, 21 years *n* = 19).

### Procedure

#### SAND Study Overview

The SAND study has four assessment waves. Waves one to three were conducted over three consecutive years and the fourth wave took place between one and three years after W3. At W1 and W3 participants attended the university twice, for the Pre-Lab session and Lab-session. At W2 data were either collected at participants’ own primary schools in classrooms or at the university in lecture rooms. Wave four was again conducted at the university during a modified Pre-Lab session (excluding instructions for Lab-session, see following section). These sessions were supervised by SAND study researchers.

In the Pre-Lab session participants completed a battery of assessments including questionnaires on a pc, tests to assess cognitive capacities, a pictorial test measuring pubertal development and a sentence completion test to measure psychosocial development. In addition, the purpose of this session was to familiarize participants with the nature of the public speaking task that took place during the Lab-session (i.e., the Leiden Public Speaking Task (PST); Westenberg et al. 2009). Participants were encouraged to prepare for the speech as they would for a school presentation. One week after the Pre-Lab session participants returned for the Leiden-PST. This session involved participants giving a 5 min speech on the type of films they like and/or dislike and explaining why, using an example of a film to illustrate their reasoning. Participants spoke in front of a pre-recorded audience consisting of four boys and four girls, matched to the participant’s age, and a female teacher. The audience was filmed in a classroom setting. The recording begins with an empty classroom and after 10 s the pupils and teacher walk into the room, take their seats and then look into the camera. The audience was projected life-size onto a screen and without a soundtrack. For more details see Westenberg et al. (2009).

#### Collection of Social Anxiety and Other Variables

Data used for social anxiety trajectory analyses were collected from participants at each assessment wave. Collection of the social anxiety questionnaire always took place at the university, except for participants attending one of the primary schools at W2 when the questionnaire was completed at school.

All other variables were measured at W1. One of the child self-report variables, negative interpretations, was collected during the Pre-Lab session. The measures of self-evaluation of performance and self-focused attention were collected during the Lab-session as these variables referred to the participants’ behavior and cognitions during the Leiden-PST. Specifically, self-evaluation of performance evaluation and self-focused attention questionnaires were filled in immediately after the speech had finished.

Independent observer report of participants’ social skills and nervousness during the speech was collected after all 331 participants had completed W1. Three psychology students acted as observers, blind to the SAND study’s hypotheses, and viewed recordings of participants’ speeches on a life-size screen (1.5 m by 2 m) in a laboratory. Observers rated the speeches independently of each other using the observer version of the Performance Questionnaire (PQ; see Measures section).

Parent and teacher reported variables were collected by means of a questionnaire booklet (one for parents and one for teachers). We asked the primary caregiver to complete the parent questionnaire booklet and the participant’s mentor to complete the teacher booklet. This information was collected between one month prior to and 5 months following the Pre-Lab session. In total, 266 participants had complete data for the parent and 318 participants for the teacher variables.

### Measures

#### Social Anxiety (Self-report)

The Dutch translation of the Social Anxiety Scale for Adolescents (SAS-A; La Greca and Lopez 1998) provided the measure of social anxiety. This 22-item instrument contains 18 descriptions of social evaluative fears and the experience of social avoidance and distress in social situations (e.g., “I worry about what other kids think of me” and “I get nervous when I meet new kids”) and 4 filler items. Respondents are asked to rate each item according to the degree to which the item “is true for you” (1 = *not at all*, 5 = *all the time*). Total scores on the SAS-A can range between 18 and 90. The SAS-A has good internal consistency (La Greca and Lopez 1998) and in the four waves of the present study Cronbach’s alpha was not lower than 0.93.

#### Negative Interpretations (Self-report)

Negative interpretations were measured with the Adolescents’ Interpretation and Belief Questionnaire (AIBQ; Miers et al. 2008). The AIBQ contains five social and five nonsocial ambiguous situations and participants rate three interpretations of the situation, positive, negative and neutral separately according to how likely it is that it would pop up in their mind. Each interpretation is rated on a 5-point Likert scale (1 = *Doesn’t pop up in my mind*, 3 = *Might pop up in my mind*, 5 = *Definitely pops up in my mind*). Because previous research (Miers et al. 2008) has shown that negative interpretations of ambiguous social situations best discriminate high from low socially anxious adolescents only data from this subscale was used. Cronbach’s alpha in the current sample was 0.73.

#### Self-focused Attention (Self-report)

The degree to which participants engaged in self-focused attention during the speech was measured with a Dutch translation of the Focus of Attention Questionnaire (FAQ; Woody et al. 1997). An example item is “I was focusing on my internal bodily reactions (for example, heart rate).” Each item is rated on a 5-point scale ranging from *not at all* (1) to *totally* (5) according to how much the participant’s attention matched the item description. Previous studies have reported acceptable internal consistency in adult (Woody et al. 1997) and youth samples (Hodson et al. 2008). We used a 4-item version of the self-focus subscale (Miers et al. 2011a) with an internal consistency of α = 0.61 in the current sample.

#### Self-evaluation of Performance (Self-report)

To measure participants’ overall evaluation of their performance during the speech the Dutch translation and adaptation of Spence et al.’s (1999) cognitive measure was used (Miers et al. 2009). The questionnaire measures both the participant’s own evaluation of their performance and expectation of evaluation from others. For the current study the question asking how scared the participant felt giving the speech was removed because this item does not reflect an evaluation of the speech performance. Items are rated using a 5-point scale (1 = lowest performance evaluation, 5 = highest performance evaluation). Internal consistency was good, α = 0.80.

#### Nervousness and Social Skills (Independent Observer Report)

An observer version of the Performance Questionnaire (PQ; Cartwright-Hatton et al. 2005) as described in Miers et al. (2009) was used to collect independent observer ratings of participants’ nervousness and social skills during the speech. This questionnaire contains 11 items that are rated on a 4-point scale ranging from *very much* (1) to *not very much* (4). Inter-rater agreement across the three observers for the nervousness and social skills subscales was high (ICC’s > 0.90; Miers et al. 2009). We averaged scores from the observers to create subscale scores. Subscales are coded so that higher scores represent greater nervousness and better social skills.

#### Neuroticism and Extraversion (Parent Report)

The Hierarchical Personality Inventory for Children (HiPIC; Mervielde & DeFruyt 2002) was completed by parents to assess participants’ personality. The HiPIC contains 144 items that assess a total of five trait domains, namely Extraversion, Benevolence, Conscientiousness, Neuroticism and Imagination. The items are worded in third person singular, refer to a specific observable behaviour and exclude personality descriptive adjectives. Items are rated on a 5-point Likert scale ranging from *barely characteristic* (1) to *very characteristic* (5). The two domains of interest in the current study are Extraversion and Neuroticism.^1^ Extraversion consists of energy, expressiveness, optimism, and shyness (reverse coded); Neuroticism consists of anxiety and self-confidence (reverse coded). These two domains are similar in content to the respective subscales in the Big Five (De Clerq et al. 2004). The HiPIC has been used extensively in different research fields, such as developmental and clinical psychology, and pediatrics, has a robust factor structure and high domain internal consistencies as documented in studies with clinical and nonclinical samples (De Clerq et al. 2004). In the current study’s sample Cronbach’s alphas were 0.82 for Extraversion and 0.85 for Neuroticism.

#### Behavioral Inhibition (Parent Report)

Behavioral inhibition was measured with the parent version of the Behavioral Inhibition Scale (BIS; Gest 1997). The parent version of the BIS provides a meaningful first impression of a child’s behavioral inhibition (van Brakel et al. 2004). We used the version containing 8 items that reflect shyness, communication, fearfulness, and smiling when talking to an unfamiliar child and an unfamiliar adult. Items are rated on a scale from *never* (1) to *always* (4). Cronbach’s alpha was 0.92.

#### Social Problems (Teacher Report)

Social problems were measured using the Conners’ Teacher Rating Scale (CTRS-R; Conners 2004) a widely used measure to screen for psychological adjustment problems. In the current study the 39-item list was used that contains six subscales, Hyperactivity-impulsivity, Perfectionism, Inattention/Cognitive Problems, Social Problems, Oppositional, and Anxious/Shy. Items are rated on a 4-point Likert scale ranging from *wholly not true/never or seldom* (0) to *completely true/very frequently* (3). The Social Problems subscale includes five items such as not being accepted by the peer group, having few friends, and poor social skills. In the current sample Cronbach’s alpha was 0.89.

#### Social Withdrawal (Teacher Report)

Teachers completed the “Vragenlijst voor Sociaal Functioneren”, the Questionnaire for Social Functioning, which consists of 8 items (Bokhorst 1990). Three items measure withdrawn behavior in pupils and are rated on a 5-point Likert scale ranging from *never* (1) to *always* (5). The specific items refer to the pupil ignoring other children, avoiding other children and participating in the activities of their age peers (recoded). Cronbach’s alpha for the withdrawn subscale was 0.79 in the current sample.

### Data Analyses

There were two main parts to the analyses. First, to identify trajectories of social anxiety we used the semiparametric group-based trajectory modeling technique, LCGM (Nagin 2005), a form of growth mixture modeling. This technique is suitable for cohort-sequential longitudinal designs (Vermunt et al. 2008). Trajectories were examined with the SAS (version 9.1) PROC TRAJ program (Jones et al. 2001). To allow for the possibility of finding a nonlinear trajectory that shows a developmental peak (e.g., Sumter et al. 2009) we modeled the data by age rather than assessment wave. A censored normal distribution was used as the basis of model estimation. Because PROC TRAJ is able to include participants who do not have data at all time points (Marmorstein et al. 2010; Mazza et al. 2010) participants who contributed at least one data point were used in the trajectory analyses. PROC TRAJ assumes that within group variability is zero (Nagin 2005). We chose this approach because allowing for within group heterogeneity requires a larger sample than we had available.

In identifying trajectories we employed the two stage procedure described by Nagin (2005). This involved first testing models of between one and six trajectories that included cubic slope terms to allow trajectory shapes to change substantially over time. The optimal number of trajectory groups is determined using the Bayesian information criterion (BIC) and the size of the trajectory groups (a minimum of 5 % for the smallest group, Andruff et al. 2009). The best fitting model is indicated by the BIC closest to zero. Once the optimal number of trajectories is selected, the second step is to determine the form of each trajectory using backward removal of nonsignificant higher-order trends, removing cubic trends first, then quadratic and, if appropriate linear trends. BIC scores were inspected during this process. Finally, once the best fitting, most parsimonious model is determined it is compared to the initial model with cubic trends for all groups. In addition, the final model’s average posterior probabilities (PP) of group membership were examined. Average posterior probabilities greater than 0.70 to 0.80 suggest that the model adequately groups participants together with homogenous patterns of change and discriminates between participants with heterogeneous patterns of change (Nagin 1999).

In the second part of the analyses, multinomial logistic regression models were used to examine which variables^2^ would discriminate between trajectories of social anxiety (Duchesne et al. 2010). We tested three models according to the categories of variables: Model 1 included the cognition variables negative interpretations, self-focused attention, and self-evaluation of performance; Model 2 included social competence variables, with independent observer evaluations of nervousness and social skill and social problems reported by teachers; Model 3 included the temperament variables of neuroticism, extraversion, and behavioral inhibition reported by parents and social withdrawal reported by teachers. Gender was included as a control variable in each model (girl = 1).

## Results

### Trajectories of Social Anxiety

**Fig. 1:**
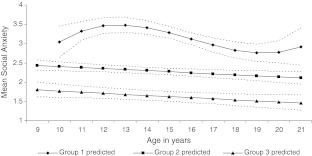
Trajectories of social anxiety in solid lines and 95 % confidence intervals in dashed lines. Group 1 = high and changing; Group 2 = moderate and decreasing; Group 3 = low and decreasing

To answer the first research question, results from initial model specification revealed that a three group model showed the best fit to the data (BIC = −1026.96) compared to models with one (BIC = −1164.69), two (BIC = −1067.45), four (BIC = −1028.82), five (BIC = −1027.53), and six (BIC = −1035.70) groups. Moreover, BIC-based model probability calculations (Nagin 1999) provided support for the three group model compared to other models.^3^ Next, the initial model with three cubic group trends was pared down and the estimated social anxiety trajectories in the final model are depicted in Fig. 1. The final three group model had a better fit to the data (BIC = −1017.37) than the initial three group with cubic trends (BIC = −1026.96) and the average posterior probability for all three groups easily satisfied the 0.80 criterion (see following section).

Starting from the top of Fig. 1, the first trajectory (9.6 %, *n* = 30, PP = 0.92, constant = 2.57, *SE* = 0.37, *p* < 0.001, linear slope = 0.56, *SE* = 0.23, *p* < 0.02, quadratic slope = −0.10, *SE* = 0.04, *p* < 0.01, cubic slope = 0.005, *SE* = 0.002, *p* < 0.02), *high and changing* social anxiety comprises youth who begin with relatively high levels of social anxiety, show an increase, followed by a decrease and then a leveling off from 18 years. The second trajectory (54.2 %, *n* = 183, PP = 0.87, constant = 2.43, *SE* = 0.06, *p* < 0.001, linear slope = -0.03, *SE* = 0.01, *p* < 0.01), *moderate and decreasing* social anxiety includes youth who begin with moderate levels of social anxiety and then show a slight decrease in anxiety scores with age. The third trajectory (36.2 %, *n* = 118, PP = 0.85, constant = 1.79, *SE* = 0.07, *p* < 0.001, linear slope = −0.03, *SE* = 0.01, *p* < 0.01), was called *low and decreasing* social anxiety, because youth in this group begin with low levels of social anxiety at age 9 and these scores decrease into early adulthood. The nonoverlapping 95 % confidence intervals displayed in Fig. 1 indicate that the trajectories are distinct (Jones and Nagin 2007). The distribution of age cohort^4^ at W1 (*χ* ^2^(16) = 17.01, *ns*) and gender (*χ* ^2^(2) = 5.33, *ns*) was similar in each trajectory.

In answer to the first research question if it is possible to identify distinct longitudinal trajectories of social anxiety across the adolescent and emerging adulthood period, 9 to 21 years, our findings show that three trajectories of social anxiety can be identified. The three trajectory groups represent high, moderate and low social anxiety levels, with increasing then decreasing high levels in the first group and a steady but small decline in the moderate and low groups. For the sake of simplicity we will refer to the trajectories as *high*, *moderate* and *low* when reporting on the relations between trajectories of social anxiety and the cognition, social competence, and temperament variables.

### Trajectories of Social Anxiety and Predictor Variables

Descriptive information for the W1 predictor variables in the whole sample and boys and girls separately is presented in Table 1. Girls had significantly higher scores than boys on the measures of negative interpretations and observer rated social skills.

**Table 1:** Descriptive statistics of predictor variables in the whole sample and by gender

Variables	Min-Max	All *M (SD)*	Boys *M (SD)*	Girls *M (SD)*	*T* boys versus girls
Cognition					
Negative interpretations	1.00–4.60	2.64 (0.81)	2.51 (0.79)	2.77 (0.82)	−2.96**
Self-focused attention	1.00–4.75	2.16 (0.75)	2.17 (0.75)	2.16 (0.74)	0.08
Self-evaluation of performance	1.33–4.67	2.90 (0.53)	2.93 (0.54)	2.88 (0.53)	0.98
Social competence					
Social performance: Nervousness	1.00–2.75	1.50 (0.30)	1.52 (0.31)	1.48 (0.29)	1.09
Social performance: Social skills	1.33–3.61	2.54 (0.37)	2.46 (0.39)	2.62 (0.33)	−4.04**
Social problems at school	0.00–2.80	0.37 (0.53)	0.45 (0.57)	0.28 (0.47)	2.80
Temperament					
Neuroticism	1.00–4.13	2.39 (0.63)	2.36 (0.63)	2.43 (0.64)	−0.89
Extraversion	1.63–4.81	3.41 (0.58)	3.40 (0.60)	3.42 (0.56)	−0.30
Behavioral inhibition	1.00–4.00	2.10 (0.64)	2.08 (0.63)	2.12 (0.65)	−0.44
Social withdrawal	1.00–4.00	2.11 (0.70)	2.15 (0.70)	2.08 (0.70)	0.84

** *p* < 0.005 (0.05/10), Bonferroni correction

For the purposes of the multinomial logistic regression analyses to answer the second research question an individual was assigned to a trajectory group based on the highest posterior probability. We chose the moderate group as reference category because it represented the largest proportion of participants and allows for comparisons with trajectories of a more extreme level (see e.g., Duchesne et al. 2008). Our main interest was to see how the high group was different from the moderate group, this latter group representing individuals with lower but not extremely low social anxiety. The highest correlation between predictor variables was −0.65 between extraversion and behavioral inhibition, hence multicollinearity was not a concern (Field 2009).^5^ Inspection of residuals and influence measures for each regression separately revealed no extreme or influential cases.

Taking the first regression model (Table 2) two of the three cognition variables contributed to the odds of following either the high or the low trajectory versus the moderate trajectory, namely negative interpretations and self-focused attention. Specifically, the probability of belonging to the high group was greater for participants with an increased tendency to have biased interpretations of ambiguous social situations (odds ratio = 3.09, *p* < 0.01) and to attend closely to one’s own feelings and behavior during a speech (odds ratio = 2.27, *p* < 0.01). Conversely, the probability of belonging to the low group was lower for participants with increased levels of negative interpretations and self-focused attention (odds ratios = 0.28, *p* < 0.01, and 0.65, *p* < 0.05 respectively).

**Table 2:** Multinomial logistic regressions predicting social anxiety trajectory group membership

	Overall model statistics		Low and decreasing		High and changing	
	*χ* ^2^(df)	Factor *χ* ^2^(df)	*B(SE)*	OR (95 % CI)	*B(SE)*	OR (95 % CI)
Model 1: Cognition	116.93(8)**					
Gender (girl)		1.43(2)	−0.28(0.27)	0.76 (0.45–1.29)	0.24(0.47)	1.27 (0.50–3.18)
Negative interpretations		70.13(2)**	−1.26(0.20)	0.28** (0.19–0.42)	1.13(0.34)	3.09** (1.59–5.98)
Self-focused attention		14.94(2)**	−0.44(0.21)	0.65* (0.43–0.97)	0.82(0.29)	2.27** (1.29–4.00)
Self-evaluation of performance		5.70(2)	0.08(0.26)	1.09 (0.65–1.81)	−1.04(0.46)	0.36 (0.14–0.88)
Model 2: Social Competence	27.26(8)**					
Gender (girl)		7.99(2)*	−0.27(0.25)	0.77 (0.47–1.26)	1.04(0.46)	2.84*(1.15–6.99)
Social performance: Nervousness		2.18(2)	−0.38(0.42)	0.68 (0.30–1.55)	0.63(0.67)	1.87 (0.50–7.02)
Social performance: Social skills		7.56(2)*	−0.56(0.34)	0.57 (0.29–1.12)	−1.54(0.63)	0.22* (0.06–0.73)
Social problems at school		11.38(2)**	−0.42(0.27)	0.66 (0.39–1.11)	0.81(0.32)	2.25* (1.20–4.22)
Model 3: Temperament	36.00(10)**					
Gender (girl)		5.37(2)	−0.32(0.28)	0.73 (0.42–1.27)	0.81(0.47)	2.25 (0.90–5.62)
Neuroticism		9.43(2)*	−0.59(0.26)	0.55* (0.33–0.93)	0.66(0.42)	1.94 (0.85–4.44)
Extraversion		1.47(2)	0.38(0.34)	1.46 (0.75–2.84)	−0.13(0.54)	0.88 (0.30–2.55)
Behavioral inhibition		0.63(2)	0.05(0.29)	1.05 (0.60–1.86)	0.36(0.46)	1.44 (0.59–3.51)
Social withdrawal		3.71(2)	−0.09(0.22)	0.92 (0.60–1.40)	0.58(0.33)	1.78 (0.94–3.37)

The Moderate and decreasing group is the comparison group for model tests and odds ratios.

An extra analysis using the low trajectory as reference group showed that, similar to the main analysis, negative interpretations, self-focused attention, social problems and neuroticism (but not social skills) differentiated the high from the low trajectory.

** *p* < 0.01; * *p* < 0.05.

The second regression model significantly predicted trajectory group membership with main effects for observer ratings of social skills, but not nervousness, and teacher rated social problems. These effects applied only to the odds of belonging to the high versus moderate trajectory. The odds ratio shows that the probability of belonging to the high trajectory was lower for participants evaluated by independent observers as socially skilled (odds ratio = 0.22, *p* < 0.05). In addition, a high level of teacher reported social problems increased the probability of belonging to the high group (odds ratio = 2.25, *p* < 0.05).

The temperament regression model showed that none of these variables contributed to the probability of belonging to the high versus moderate group. The significant effect for neuroticism shows that participants whose parent reported increased levels of neuroticism were less likely to belong to the low compared to the moderate trajectory (odds ratio = 0.55, *p* < 0.05).

Finally, gender had a main effect in the social competence model only. Girls were significantly more likely than boys to belong to the high versus the moderate trajectory. This suggests that one or more of the social competence variables acts as suppressor on gender and that with social competence level kept constant differences between boys and girls in trajectory group membership do occur.

In sum, in answer to the second research question, which of the cognition, social competence and temperament variables discriminates between trajectories of social anxiety, results indicate that negative interpretations and self-focused attention discriminate both the high and the low trajectory from the moderate trajectory. Social performance skills and social problems at school discriminate the high from the moderate trajectory, and finally neuroticism distinguishes the low from the moderate trajectory.

## Discussion

This longitudinal study is the first to examine developmental trajectories of social anxiety in a nonclinical sample aged 9 to 21 years and simultaneously test whether conceptually relevant individual level variables assessing cognition, social competence, and temperament discriminated between the trajectories. The study contributes to knowledge of the course of social anxiety during adolescence and emerging adulthood in at least two ways. First, the results support the presence of distinct trajectories of social anxiety across adolescence and provide an initial exploration of trajectories in emerging adulthood. Second, adolescents following a high social anxiety trajectory can be discriminated from peers belonging to a less anxious trajectory, based on their self-reported cognitions and observed social behavior in a laboratory task and in the classroom.

### Trajectories of Social Anxiety

In answer to the first research question, can we identify distinct longitudinal trajectories of social anxiety during adolescence and emerging adulthood, we found three trajectories: i) high and changing; ii) moderate and decreasing; iii) low and decreasing. The pattern of trajectories supports our expectation of identifying at least a high increasing and a low trajectory. As would be expected in a nonclinical sample the high and changing group represented the smallest trajectory (9.6 %). This group had an initial high level of social anxiety, at age 10, and showed a rise early in adolescence, before decreasing and then leveling off from 18 years. Our low and decreasing trajectory, including a little more than a third of the sample (36.2 %), began with a low level of social anxiety that decreased slightly over time. Although not explicitly predicted, the third trajectory of social anxiety, moderate and decreasing, represented the largest trajectory (54.2 %) that began with a moderate level of social anxiety and decreased over time. It should be kept in mind that, given the smaller number of participants in the preadolescent and emerging adulthood age groups, the pattern of trajectories identified in these stages is only a preliminary finding.

Compared to previous studies of (social) anxiety trajectories (Broeren et al. 2011; Crocetti et al. 2009; Duchesne et al. 2010; Feng et al. 2008; Marmorstein et al. 2010), our high trajectory showed a changing as opposed to a stable or increasing pattern. This could be explained by the fact that we modeled social anxiety by age and not by assessment wave as in the case of the Crocetti et al. (2009) study. As compared to the studies by Broeren et al. (2011), Duchesne et al. (2010), Feng et al. (2008) and Marmorstein et al. (2010) the changing pattern in our high trajectory could be attributable to the wider age range investigated here, which started at age 9 through 21, whereas in these previous studies the oldest group was aged 13. This highlights the benefit of investigating a wider, and older, age range, including the emerging adulthood period. Moreover, the temporary increase between ages 10 and 14 years, in an already high social anxiety level, is in line with the idea that high levels of social anxiety in childhood become more problematic in early adolescence (Inderbitzen-Nolan and Walters 2000; Rapee and Spence 2004). Early adolescence is perhaps the most difficult stage within the whole adolescent period. The identification of a low and fairly stable or slightly decreasing trajectory of anxiety seems to be characteristic of both the childhood (Broeren et al. 2011; Duchesne et al. 2010; Feng et al. 2008) and adolescent periods (Crocetti et al. 2009). Lastly, our moderate and decreasing trajectory can be considered as similar to the moderate, but stable, groups reported in both Broeren et al. (2011) and Marmorstein et al. (2010). Hence, the three trajectories of social anxiety found here, although not explicitly predicted, are broadly in keeping with previous studies of generalized or social anxiety trajectories.

### Trajectories of Social Anxiety and Relations with Cognition, Social Competence, and Temperament

The second research question asked which of the cognition, social competence, and temperament variables would discriminate between trajectories of social anxiety. Partly in line with expectations, the cognition and social competence variables discriminated the high from the moderate trajectory, but the temperament variables did not.

Findings from the cognition regression model showed that, in line with expectations, higher levels of self-reported negative interpretations and self-focused attention are related to the likelihood of following the high versus the moderate trajectory. These results are broadly consistent with, and add to previous literature documenting cross-sectional associations between social anxiety and negative interpretations and social anxiety and self-focused attention (Hodson et al. 2008; Miers et al. 2011b). Self-evaluation of performance did not reach significance in the multivariate tests. Performance evaluation is less strongly related to trajectories of social anxiety compared to the other two cognition variables. However, the odds ratio and confidence intervals for the high trajectory indicate that this variable may also be of importance in discriminating the high from moderate trajectory. The finding that negative interpretations and self-focused attention differentiated between all three trajectories implies a linear relationship between these cognition variables and social anxiety. Negative cognitions are not specific to a high social anxiety group but co-vary with social anxiety over the whole range.

Second, and confirming expectations regarding social competence, we found that observer rated social skills and teacher rated social problems specifically differentiated the high from the moderate trajectory. Poorer social skills during a speech task and greater social problems at school, such as few friends and not being accepted by peers, are linked to the high trajectory. These findings are in line with the social skills deficit hypothesis of social anxiety (Miers et al. 2010) and extend the extant cross-sectional literature on first time peer judgments and outcomes from long-term interactions (e.g., Inderbitzen-Nolan et al. 2007; Spence et al. 1999). In contrast, the social competence variables did not differentiate the low from the moderate trajectory, suggesting a comparable level of social skills and social problems in these two trajectories.

Our finding that observer rated nervous behavior was not related to the chances of following the high trajectory is perhaps quite surprising when considered in light of an expected high state anxiety of socially anxious persons during a speech task (Mauss et al. 2004) and the assumption of cognitive models (Clark and Wells 1995; Rapee and Heimberg 1997) that the visibility of one’s nervousness is a key concern of socially anxious persons. Nevertheless, this finding is in keeping with studies that did not find a difference in observed nervousness between high socially anxious youth and a control group (e.g., Cartwright-Hatton et al. 2005; Miers et al. 2009). Possibly, it is not the actual observable nervousness that is important to the development of high social anxiety, but the interpretation of anxious symptoms during a social stressor. The cognitive models describe how socially anxious persons closely monitor their physical symptoms of anxiety and interpret these symptoms in a distorted and exaggerated manner, for example a slight warm feeling in the cheeks is interpreted as visible blushing (Miers et al. 2011b). In turn, these interpretations reinforce the negative cognitions concerning how other people judge their behavior or appearance in the situation. Hence, a more important variable might be socially anxious persons’ perception of their anxious symptoms.

Third, in contrast to expectations none of the temperament variables differentiated the high from the moderate trajectory. The likelihood of belonging to the high trajectory was not related to parent reported neuroticism, extraversion or behavioral inhibition nor to teacher reported social withdrawal, as predicted on the basis of previous literature (e.g., Beidel et al. 1999; Chronis-Tuscano et al. 2009; Ladd 2006). Neuroticism was related to the likelihood of belonging to the low versus moderate trajectory, with lower neuroticism in the low trajectory, whereas neuroticism was found equally in the moderate and high trajectories. Although somewhat surprising these results, particularly for behavioral inhibition and social withdrawal, are consistent with some existing trajectory studies in which these variables did not differentiate between high or increasing trajectories and lower trajectories of anxious (Feng et al. 2008) and depressive symptoms (Mazza et al. 2010). It is possible that the moderate and high trajectories are characterized by a similarly high level of neuroticism and behavioral inhibition in childhood but due to compensatory factors such as social support and social skills, the moderate group did not develop high social anxiety in adolescence. Alternatively, for behavioral inhibition a measure collected in early childhood, such as maternal report or behavioral observation (Chronis-Tuscano et al. 2009), may be a more valid measure of this temperamental style and therefore show a stronger relation to a high social anxiety trajectory.

The present study has a number of limitations. First, although the sample size of the present study was sufficient (Nagin 2005) for the purposes of the study and comparable to previous studies in this area (e.g., Feng et al. 2008), the findings should be replicated in a larger sample (at least 500) to draw more reliable conclusions. Second, the limited sample size of the high trajectory also meant that we could not examine the relative importance of cognitive, social competence, and temperament variables in one model. Also, this might have reduced the power to detect relations with the chosen variables and contributed to the wider confidence intervals of this group at the youngest and oldest age groups. Third, in this study’s cohort design measurement of the cognitive, social competence, and temperament variables did not precede the start of the trajectories. This means that the relations reported between these variables and the different trajectories do not demonstrate causality or their actual temporal ordering.

### Suggestions for Future Research, Clinical Implications and Conclusions

Because of the limited sample size we examined single variables only rather than interactions between variables. It is plausible that greater predictive power would be gained by studying interactions. As suggested in recent literature it is likely that temperament variables would differentiate a high social anxiety trajectory from lower trajectories when considered in interaction with environmental level variables such as parenting or peer interactions (Degnan et al. 2010; Hayward et al. 2007). For example, behavioral inhibition in and of itself was not related to high social anxiety during adolescence, but in interaction with poor social skills it could lead to a particularly poor outcome. On the other side, an inhibited child may be protected from following a pathway with problematic social anxiety levels by their good social skills, which ensure access to the peer group. In addition, other individual and environmental level variables not included in the present study may be related to the emergence of social anxiety during adolescence, either alone or in interaction. For example, attributes of a child’s friendships (quality and number), parental influences (parenting style, transmission of negative cognitions), and attention biases would be a useful addition. Further studies investigating social anxiety trajectories are needed in order to explore these avenues and to corroborate the findings of the present study, in particular by collecting information about single variables from different informants.

Taking the findings of this study together some implications for clinical intervention and prevention may be put forward. The pattern of trajectories suggests that a high and potentially problematic level of social anxiety develops in children around the ages of 9 to 10 years. Of course we cannot make firm conclusions about the most problematic age in terms of social anxiety levels because we do not have data spanning childhood and adolescence. Nevertheless, it seems feasible to suggest that prevention strategies should be implemented before pre to early adolescence and diagnostic assessment should include a range of instruments measuring not only social anxiety but also cognition and social competence. High risk children could then be taught social skills and given the opportunity to practice these skills in situations with unfamiliar peers to improve their effectiveness in social situations at school and in first-time interactions. It is imperative to improve a child’s actual social skills as well as modify negative cognitions in intervention programs as without an improvement in skills that helps to elicit positive feedback from others, the negative cognitions will simply be reinforced (Miers et al. 2011b).

In conclusion, the present study adds to the existing social anxiety literature by identifying, for the first time, social anxiety trajectories during the adolescent period. The findings suggest that cognition and social competence variables may be reliably used to identify adolescents at risk of belonging to a high social anxiety trajectory. Together with previous research (Inderbitzen-Nolan et al. 2007; Miers et al. 2010) our results point to the importance of improving social skills in the treatment of social anxiety in order to increase the chances that a child will seek access to their peer group and subsequently receive positive responses from their peers. At the same time, modifying the tendency to self-focus during stressful social situations and negatively interpreting ambiguous social cues could serve to reduce fear of social situations.

## Footnotes

1 For the SAND study one item from the extraversion subscale and 3 items from the neuroticism subscale were modified slightly because these items contained Belgian-Dutch expressions that are not widely used in the nonBelgian Dutch speaking population. These items were adjusted with permission from F. De Fruyt, one of the original authors (May 2006, personal communication). Item 51 was changed from “heeft energie te koop” to “heeft energie te over”; item 58 was changed from “heeft de neiging om te wenen bij tegenslag” to “heeft de neiging om te huilen bij tegenslag”; item 94 was changed from “heeft schrik om fouten te maken” to “is bang om fouten te maken”; item 130 was changed from “heeft vlug schrik” to “is vlug bang”.

2 In the multinomial logistic regressions the cognition, social competence, and temperament variables are statistical predictors of trajectory membership. Hence, the term ‘predictor’ is used in the Results and the interpretation of the predictor variables in relation to trajectories of social anxiety is addressed in the Discussion.

3 The BIC-based model probability calculations are available from the first author.

4 No participant from the 9 year old cohort was allocated to the high and changing trajectory.

5 The full inter-correlation matrix is available from the first author.
